# PARP inhibitors in testicular germ cell tumors: what we know and what we are looking for

**DOI:** 10.3389/fgene.2024.1480417

**Published:** 2024-11-29

**Authors:** Sara Parola, Christoph Oing, Pasquale Rescigno, Salvatore Feliciano, Francesca Carlino, Luca Pompella, Antonella Lucia Marretta, Irene De Santo, Martina Viggiani, Margherita Muratore, Bianca Arianna Facchini, Jessica Orefice, Eleonora Cioli, Francesca Sparano, Domenico Mallardo, Ugo De Giorgi, Giovannella Palmieri, Paolo Antonio Ascierto, Margaret Ottaviano

**Affiliations:** ^1^ Medical Oncology Unit, Ospedale Ave Gratia Plena, ASL Caserta, San Felice a Cancello, Italy; ^2^ Translational and Clinical Research Institute, Centre for Cancer, Newcastle University, Newcastle Upon Tyne, United Kingdom; ^3^ Medical Oncology Unit, Ospedale San Giuseppe Moscati, ASL Caserta, Aversa, Italy; ^4^ Department of Medical Oncology, IRCCS Istituto Romagnolo per lo Studio dei Tumori (IRST) “Dino Amadori”, Meldola, Italy; ^5^ Department of Precision Medicine, Università degli Studi della Campania “Luigi Vanvitelli”, Naples, Italy; ^6^ Department of Melanoma, Cancer Immunotherapy and Development Therapeutics, Istituto Nazionale Tumori IRCCS Fondazione G. Pascale, Naples, Italy; ^7^ Rare Tumors Coordinating Center of Campania Region (CRCTR), Naples, Italy

**Keywords:** testicular tumors, germ cell tumors, cisplatin resistance, PARP inhibitors, DNA damage response, homologous recombination repair

## Abstract

Testicular germ cell tumors (TGCTs), the most common malignancies affecting young men, are characterized by high sensitivity to cisplatin-based chemotherapy, which leads to high cure rates even in metastatic disease. However, approximately 30% of patients with metastatic TGCTs relapse after first-line treatment and those who can be defined as platinum-refractory patients face a very dismal prognosis with only limited chemotherapy-based treatment options and an overall survival of few months. Hence, to understand the mechanisms underlying cisplatin resistance is crucial for developing new treatment strategies. This narrative review explores the potential role of PARP inhibitors (PARPis) in overcoming cisplatin resistance in TGCTs, starting from the rationale of their ability to induce DNA damage in cells with homologous recombination repair (HRR). Thus far, PARPis have failed to show meaningful clinical activity in platinum-refractory TGCT patients, either alone or in combination with chemotherapy. However, few responses to PARPis in TGCTs have been detected in patients with BRCA1/2, ATM or CHEK2 mutations, reinforcing the idea that patients should be optimally selected for tailored treatments in the era of personalized medicine. Future preclinical and clinical research is needed to further investigate the molecular mechanisms of cisplatin resistance and to identify novel therapeutic strategies in resistant/refractory TGCTs patients.

## 1 Introduction

Testicular germ cell tumors (TGCTs) represent the most common malignancies in young men (aged 15–44 years) (Nappi et al., 2018).

TGCTs are histologically divided into seminomas (S) and non-seminomas (NS) and patients with metastatic disease are classified according to the International Germ Cell Cancer Collaborative Group (IGCCCG) risk classification (IGCCCG 1997; Beyer et al. JCO 2021; Gillessen et al. JCO 2021). TGCTs are characterized by high sensitivity to cisplatin-based chemotherapy which achieves high cure rates even in metastatic stages (Mead and Stenning, 1997; Gillessen et al., 2021).

Generally, about 30% of patients with metastatic TGCTs relapse despite stage-adapted first-line chemotherapy, of which 50% can be cured with salvage treatment (McHugh and Feldman, 2018).

Patients relapsing during or at least after two lines of platinum-based chemotherapy are considered as platinum-refractory. Platinum-refractory patients face a very dismal prognosis with only limited chemotherapy-based treatment options and an overall survival of only a few months (Honecker et al., 2018). To date, no targeted treatment approach has shown reasonable clinical activity in refractory TGCT patients, despite many clinical phase II trials assessing agents such as tyrosine kinase inhibitors, immune checkpoint inhibitors or antibody-drug conjugates, i.e., brentuximab vedotin (Oing et al., 2016; Kalavska et al., 2020). Hence, novel treatment approaches are needed to improve the outcomes of patients with platinum-refractory disease.

Understanding the mechanism of action of cisplatin and cisplatin-resistance is pivotal to guide the way for identifying putative molecular treatment targets and inform future clinical trials (Rescigno et al., 2020). The main mechanism of action of platinum agents is to cause multiple types of DNA damage via formation of intra- and inter-strand cross-links (ICLs) through platinum-DNA adduct formation (Caggiano et al., 2021).

ICLs covalently link the two strands among the DNA double helix, which blocks replication and transcription processes and, if ICLs remain unrepaired, ultimately induce DNA double-strand breaks (DSBs) (Cavallo et al., 2012).

In normal cells DSBs are mainly repaired through homologous recombination repair (HRR), a process where DSBs and inter-strand crosslinks are repaired using the sister chromatid as a template (Ciccia and Elledge, 2010).

Functional HRR requires various proteins, of which BRCA1 and BRCA2 are among the key facilitators of successful DSB repair (Pellegrino et al., 2020).

Inactivating mutations of the *BRCA1* or *BRCA2* genes, among others, induce HRR deficiency (HRD) forcing cells to utilize alternative, error-prone DSB repair pathways such as non-homologous end joining (NHEJ) (Schettini et al., 2021). *BRCA1/2* mutation-associated HRD sensitizes cancer cells to poly (ADP-ribose) polymerase (PARP) inhibitors (PARPi). PARP comprise of a family of nuclear enzymes, which are involved in the recognition and repair of DNA single-strand breaks (SSBs) (Messina et al., 2020).

They act by trapping PARP molecules causing the dysfunctional DNA SSB repair, leading to the accumulation of replication-associated DSBs in cancer cells. Therefore, when HRR is constitutionally dysfunctional, as in BRCA mutant tumors, according to the concept of synthetic lethality, HR-deficient tumor cells are extraordinarily sensitive to PARPis. As a result, in case of other events that impair DNA damage, this is likely to become permanent, with progressive accumulation of DNA lesions that ultimately leads cancer cell death (Brown et al., 2017).

**FIGURE 1:**
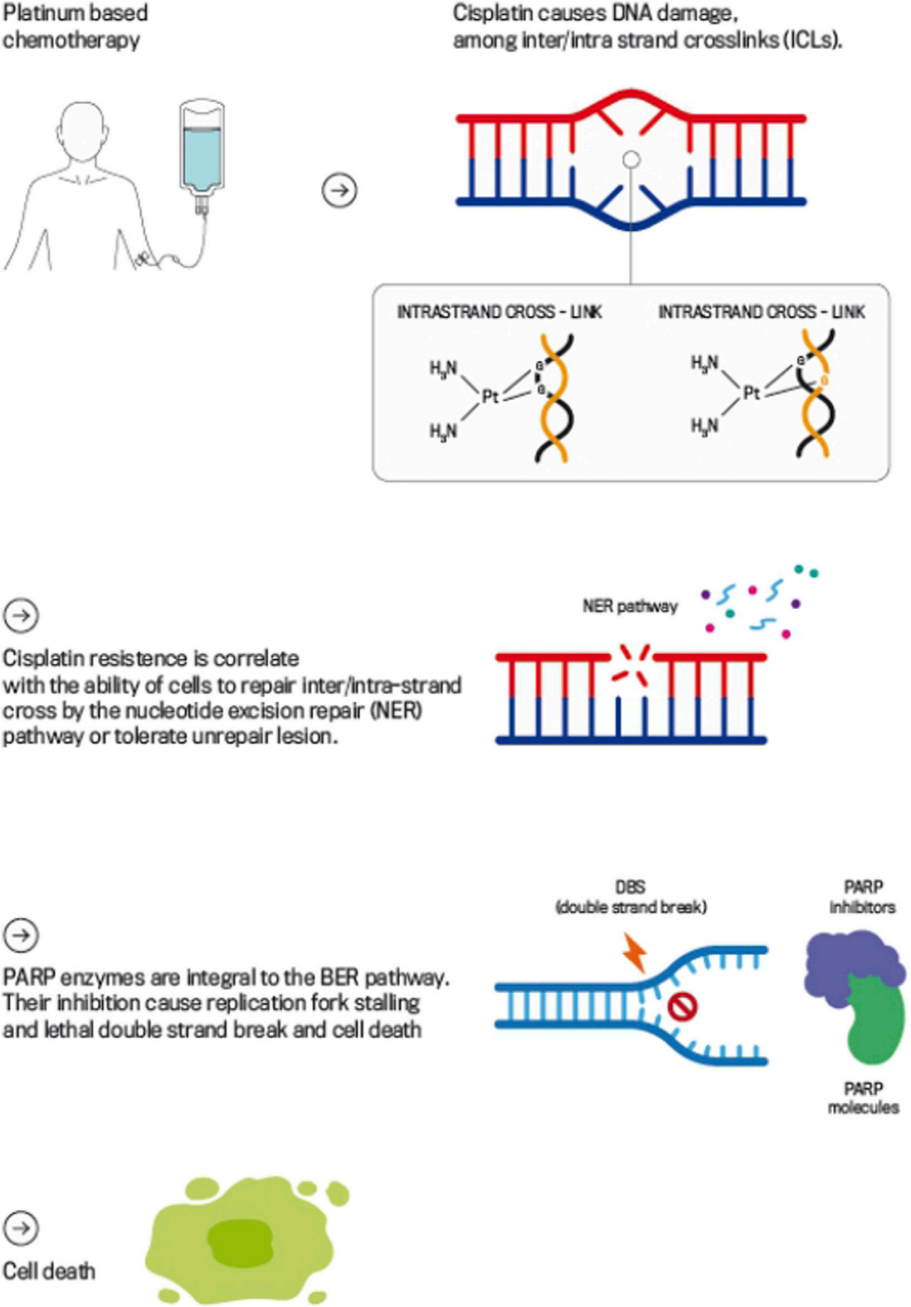
Mechanism of Action of Platinum-based Chemotherapy and PARP Inhibitors in DDR. This figure illustrates how platinum-based chemotherapy, such as cisplatin, induces DNA damage by forming intra- and inter-strand cross-links that obstruct DNA replication and transcription, leading to DSBs if left unrepaired. The figure also highlights the role of PARPi, which block the PARP enzyme’s function in repairing DBs.

Given the importance of ICLs in cisplatin-induced cytotoxicity, it is assumed that the extreme sensitivity of TGCTs to cisplatin results, at least in part, from impairment of one or more steps of the ICL repair mechanism. Therefore, DNA repair mechanisms play a pivotal role in cellular tolerance to cisplatin by passing or removing ICLs (Räschle et al., 2008; Bhagwat et al., 2009; Nakanishi et al., 2011) (Figure 1).

This intrinsic property of TGCTs may represent a potential target for treatment of tumors which acquire cisplatin resistance.

The use of PARPis in cisplatin resistant TGCTs is an area of active research.

Considering the urgent clinical need to better determine the mechanisms behind cisplatin resistance in TGCTs and to identify new attractive therapies, the aim of this narrative review is to explore and discuss the potential role of PARPis in platinum-refractory TGCT management.

## 2 Mechanisms of signal and/or DNA repair: the DNA damage response

Living organisms are continuously exposed to a myriad of DNA damaging agents that can impact health and modulate disease-states (Chatterjee and Walker, 2017). DNA repair and DNA damage signaling pathways are critical for the maintenance of genomic stability. Defects within these mechanisms can contribute to tumorigenesis. However, they also render cancer cells vulnerable to DNA injury.

Cells have evolved several pathways made by proteins, involved in DNA damage signaling and/or repair, which are collectively defined as the DNA Damage Response (DDR) (Pearl et al., 2015).

Nucleotide excision repair (NER) is one of the major pathways responsible for repairing single-strand breaks (SSBs) and is particularly significant for addressing complex lesions, such as bulky adducts and interstrand crosslinks (ICLs) induced by agents like platinum compounds (Christmann et al., 2003).

NER operates by recognizing and excising damaged DNA segments, followed by the synthesis of new DNA to replace the excised strand. This process involves two sub-pathways: global genomic repair (GGR) and transcription-coupled repair (TCR). GGR addresses all repairable lesions throughout the genome, while TCR specifically targets the transcribed DNA strand in actively expressed genes.

NER relies on eight core genes (*XPA*, *ERCC3/XPB*, *XPC*, *ERCC2/XPD*, *XPE/DDB1*, *ERCC4/XPF*, *ERCC5/XPG*, and *ERCC1*). The *XPA* protein, part of the xeroderma pigmentosum complementation group A, plays a crucial role in verifying DNA lesions and assembling the NER incision complexes. Incision is carried out by two structure-specific nucleases, *XPF* and *XPG*; *XPF* creates a complex with the excision repair cross-complementation group 1 (*ERCC1*) protein, which is catalytically inactive, but essential for targeting *XPF* to various substrates, thus regulating its activity and availability (Spivak, 2015).

The efficacy of NER in repairing cisplatin-induced ICLs is crucial for stabilizing the DNA damage caused by chemotherapy. However, proficient NER may lead to enhanced DNA repair, which could consequently reduce the effectiveness of the chemotherapy (Hanawalt et al., 2003).

Interestingly, Cierna et al. reported a link between elevated *XPA* expression in primary GCTs and poorer patient outcomes. Higher *XPA* levels are particularly common in patients with advanced disease and unfavorable prognostic features, suggesting that increased *XPA* may contribute to cisplatin resistance, leading to tumor dissemination and disease progression. This association between *XPA* expression and cisplatin response observed in patient samples was also detected in GCT cell lines, highlighting the potential of *XPA* as a biomarker for both chemo-resistance and disease severity in GCTs (Cierna et al., 2020).

The two main DSB repair pathways are HRR and NHEJ. The HRR pathway uses homologous DNA from the sister chromatid as a template during the late S and G2 phases of the cell cycle. Three processes are involved: double-strand break recognition (DSBR); synthesis-dependent strand annealing (SDSA) and break-induced replication (BIR) (Christmann et al., 2003). BRCA1 and 2 are two essential proteins for these processes (Underhill et al., 2011). BRCA1 acts at an early HRR mediator promoting the end-resection at DSB tails and, at a later step, to recruit PALB2, which promotes BRCA2 chromatin localization. BRCA2 promotes loading of RAD51 recombinase to form a RAD51-ssDNA filament, which is essential for HRR initiation (Prakash et al., 2015). Dissimilar from HR, NHEJ does not require a homologous template for the repair of DSBs and is therefore active throughout the cell cycle. As NHEJ directly ligates DSB ends without recovery of the lost genetic material it is thus error-prone than HRR and associated with a greater probability of genomic instability (Rose et al., 2020). Normal cells preferentially repair DSBs via the gene conversion sub-pathway of HRR (error free), but when there is a loss of function in HRR, e.g., trough mutations of BRCA1, BRCA2 or other related genes, cells are compelled to repair DSBs via NHEJ or the single strand annealing sub-pathway of HR, both mechanisms being prone to error (Underhill et al., 2011).

DDR mechanisms maintain genomic integrity and stability by restoring DNA damage arising from intracellular and extracellular stressors. If left unresolved, the cell evokes a programmed cell death pathway. Indeed, the specific activation or inactivation of these factors in various cancers or the development of corresponding inhibitors or activators represent a recent hot spot of cancer therapy research.

## 3 Mechanism of action of PARP inhibitors (PARPis)

PARPis are a class of anti-cancer drugs which block the enzymatic activity of PARP molecules (Rose et al., 2020).

PARP enzymes constitute a large family of 17 proteins (Amé et al., 2004), of which PARP1 and PARP2 are involved in intracellular DDR and facilitation of DNA repair (Dziadkowiec et al., 2016).

When PARP1 and PARP2 recognize the single strand DNA damage, their zinc finger DNA-binding domain can open the chromatin and catalyze the transfer of ADP-ribose to themselves (auto-PARylation) and other target proteins (PARylation). PARylation triggers the release of bound PARP from DNA, thereby facilitating the recruitment of other DNA repair factors (Satoh and Lindahl, 1992).

Hence, PARPis compete with nicotinamide (NAD+) for the catalytically active site of PARP molecules (Rose et al., 2020); more importantly, they trap PARP1 on the damaged DNA, resulting in stalled replication forks and subsequent formation of replication-induced DSBs, which can only be repaired via the HRR pathway (Murai et al., 2012; Gourley et al., 2019).

HRR is the fundamental pathway that enables error-free repair of DSBs. It relies on several proteins including BRCA1 and BRCA2, proteins of the Mre11-Rad50- Nbs1 (MRN) complex, CtIP, MRE11, RAD51, ATM, H2AX, PALB2, RPA, RAD52, and the Fanconi anemia pathway proteins (Vergote et al., 2022).

These molecules are interconnected in various DNA repair pathways and their mutations can contribute to a higher risk of cancer development through resulting genomic instability.

Tumors deficient in the HRR pathway, HRD, exhibit increased sensitivity to PARPis, particularly in BRCA1/2 deficient tumors. Conversely, HRP tumors, gain limited benefit from PARPi (Bonadio and Estevez-Diz, 2021; Stewart et al., 2022).

HRD cells exhibit increased sensitivity to PARPis, which has driven the development of these inhibitors for treating BRCA1/2-deficient tumors. The critical mechanism is synthetic lethality: HRD cells, the inability to repair these DSBs leads to either apoptosis or mitotic catastrophe, resulting in cell death (Lord and Ashworth, 2017).

This synthetic lethality is particularly relevant in cells with BRCA gene loss of function, where PARP inhibition by PARPis leads to cell death (Farmer et al., 2005). In normal cells, the loss of a single repair pathway does not necessarily cause cell death due to compensatory mechanisms that maintain cell survival. However, in cancer cells with HRD, this vulnerability can be exploited therapeutically.

**FIGURE 2:**
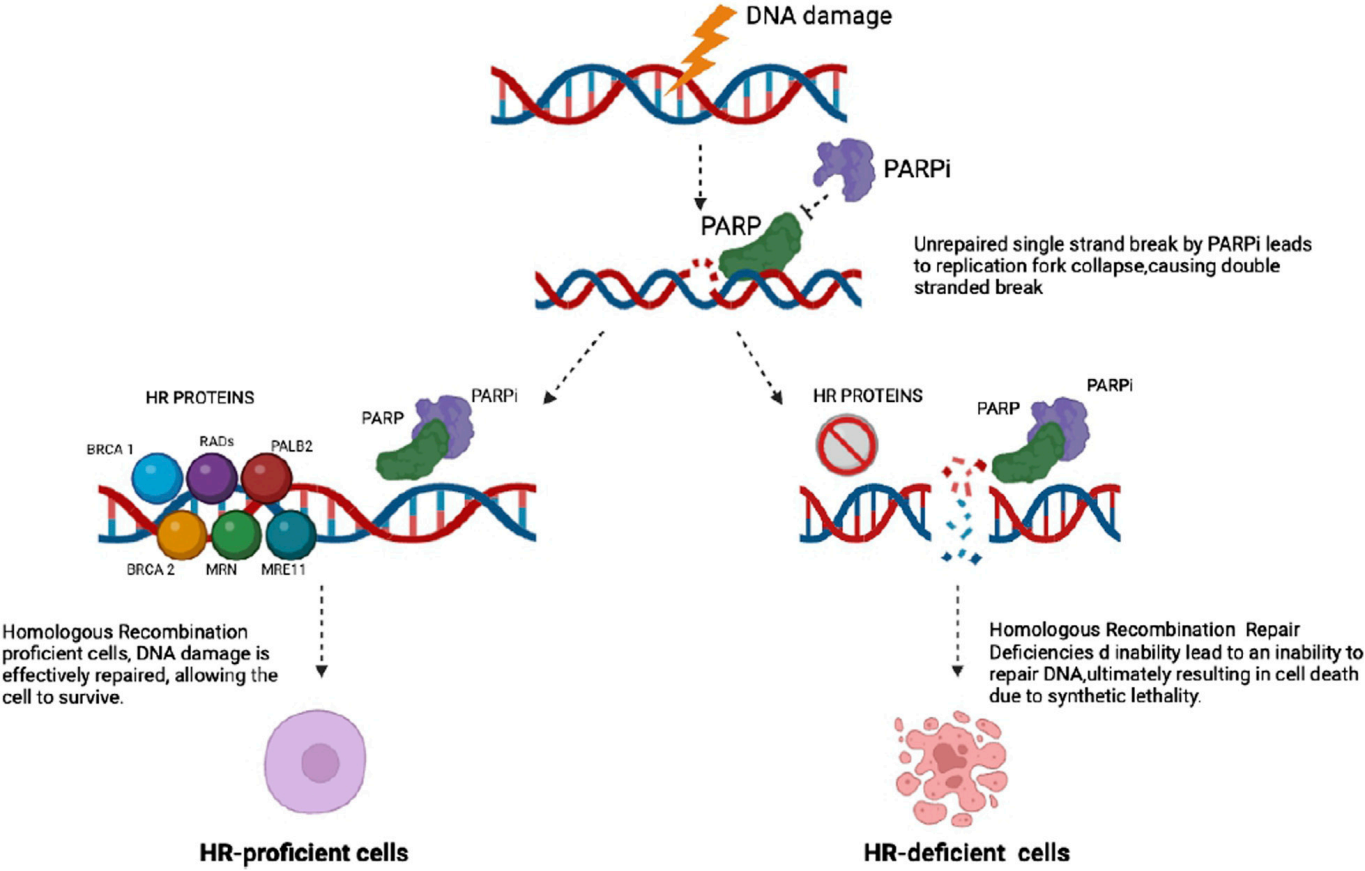
PARP inhibitors block the repair of existing DNA damage, leading to an accumulation of single strand DNA breaks (SSBs). When these SSBs occur, PARP is activated and binds to the damaged sites, attempting to facilitate repair. This binding can result in the production of DNA-protein crosslinks. Consequently, replication forks may collapse, resulting in the accumulation of double strand breaks (DSBs). In cells proficient in homologous recombination (HR), these DSBs are effectively repaired through the HR pathway. In contrast, HR-deficient tumor cells lack this repair capability, resulting in unresolved DNA damage that ultimately leads to cell death.

PARPis have thus become the standard of care for several cancers harboring BRCA mutations, including breast, ovarian, pancreatic, and prostate cancers. The development and clinical application of PARPis underscore their pivotal role in targeting cancer-specific genetic aberrations and enhancing treatment efficacy (Figure 2).

## 4 Exploring the synergistic potential of PARPis and platinum chemotherapy in TGCTs

Combining PARP inhibitors (PARPis) with other DNA-damaging agents represents a strategic approach to improve cancer treatment efficacy. Platinum-based chemotherapy, which induces cell death by adding alkyl groups to DNA bases and creating ICLs repaired by the NER pathway, is particularly relevant. In many tumor cells, key NER proteins are under-expressed, leading to insufficient repair of cisplatin-induced crosslinks and increased sensitivity to cisplatin (Rose et al., 2020).

Previous studies in testicular tumors (i.e., TGCT) have shown low NER activity in TGCT cell extracts and low expression of several key NER proteins (Köberle et al., 1999; Welsh et al., 2004). These intrinsic NER defects are correlated with high cisplatin sensitivity and a high cure rate among TGCT patient (Masters and Köberle, 2003).

Moreover, defects in DSB repair mechanisms, particularly HRR, can exacerbate the cytotoxic effects of platinum agents. If ICLs are left unrepaired due to deficient HRR, it can lead to collapsed replication forks and further accumulation of DSBs, compounding cellular damage (de Vries et al., 2020).

PARPis can work synergistically with platinum-based chemotherapy due to synthetic lethality: PARPis inhibit the SSBs, while platinum agents introduce SSBs and ultimately DSBs (Ang et al., 2010). This dual action disrupts multiple DNA repair pathways, particularly in tumors with heightened sensitivity to platinum-based therapies. The therapeutic efficacy of PARPis is particularly evident in patients harboring BRCA mutations, where the tumor’s intrinsic sensitivity to platinum treatment can enhance the activity of PARPis (Fong et al., 2010).

While the combination of platinum compounds and PARPis is still under investigation and not yet approved, emerging evidence supports their potential synergistic effects (Michels et al., 2013; Bhattacharjee et al., 2022; Frenel et al., 2022; Valenza et al., 2023).

It could be intuitive to argue that this synergy could be particularly beneficial in enhancing treatment outcomes in cases where cisplatin-based chemotherapy alone has failed to elicit the expected response.

Despite some concerns about potential cross-resistance induced by platinum pre-treatment, the concept warrants further investigation.

## 5 The rationale for PARPis in TGCTs

Patients with TGCTs receive cisplatin-based chemotherapy depending on their histology (seminoma or non-seminoma), disease stage and IGCCCG risk profile (Mead and Stenning, 1997; Gillessen et al., 2021).

Cisplatin based chemotherapy induces long-term remissions in approximately 80% of patients with advanced TGCTs treated with first-line combination chemotherapy, while the percentage drops to 50% treatment responses at first relapse/progression (Loehrer et al., 1998; Albers et al., 2015; Patrikidou et al., 2023). Indeed, patients, who relapse after initial chemotherapy with cisplatin, require second-line therapy to be cured. They can undergo either conventional salvage cisplatin-based chemotherapy or high-dose chemotherapy (HD-CT) (Patrikidou et al., 2023).

Results from the randomized phase III trial TIGER, which compared four cycles of SD-CT vs. sequential HD-CT will show, which approach is the best salvage strategy (ClinicalTrials.gov Identifier: NCT02375204).

Patients who progress during or within 1 month after completion of the initial platinum-based chemotherapy, or patients who relapse/progress after the second-line platinum-based treatment, are considered “platinum-refractory” and they have an extremely poor prognosis with long-term survival achieved in less than 5% of cases (De Giorgi et al., 2006; Lorch et al., 2011).

In these cases, there is no consensus on the optimum therapeutic strategy to adopt for achieving disease remission. HD-CT with TI-CE schedule (paclitaxel [T] plus ifosfamide [I] followed by high-dose carboplatin [C] plus etoposide [E] with autologous stem-cell support) should be considered as first option in these patients as SD-CT cannot achieve long-term disease control in this highly unfavorable group of patients [57]. According to a large retrospective analysis of almost 1,600 cases, HD-CT as first salvage treatment led to a 10%–15% improved survival probability and was the only successful treatment in patients with platinum-refractory disease (Lorch et al., 2011).

Nevertheless, in case of HD-CT failure or in patients for whom HD-CT is not feasible, the identification of new drugs and/or new combinations represents an urgent clinical, unmet need (De Giorgi et al., 2006).

Several chemotherapeutic agents have been investigated in patients with cisplatin-refractory TGCTs (i.e., paclitaxel, gemcitabine or oxaliplatin alone or in combination or oral etoposide) (Sandler et al., 1998; Bokemeyer et al., 1999; Einhorn et al., 1999; Kollmannsberger et al., 2002).

The highest response rate of 51% was reported for the triplet combination of gemcitabine, oxaliplatin and paclitaxel (GOP), but long-lasting remission are rarely achieved (Oing et al., 2018).

Due to the rarity, heterogeneity, and lack of patient selection or biomarkers for platinum-refractory illness, virtually all early phase trials evaluating molecularly targeted therapies have not yet demonstrated clinically relevant activity (Oing et al., 2016).

Thus, the investigation of the DDR pathway as a clinical target could be promising, also in cisplatin resistant testicular cancers (de Vries et al., 2020).

The mechanism of cisplatin resistance occurs due to an increase ability to repair DNA damage or an acquired ability to tolerate unrepair DNA lesion (Országhová et al., 2022).

In TGCT cisplatin resistance mechanisms can be classified into pre-target, on-target, and post-target mechanisms. Pre-target mechanisms involve reduced cisplatin uptake and enhanced detoxification by cellular components like glutathione. On-target resistance is linked to defective DNA repair pathways, such as diminished mismatch repair (MMR) and nucleotide excision repair (NER). Post-target resistance involves downstream alterations in apoptosis signaling pathways, particularly those involving p53 and related proteins. Additionally, epigenetic changes, such as DNA methylation, play a significant role in cisplatin resistance, influencing both DNA repair and transcriptional responses to damage. Overall, these mechanisms underscore the complexity of cisplatin resistance and highlight the need for further research to develop effective tailored therapeutic strategies (Skowron et al., 2021)

Considering these resistance mechanisms, PARPis may offer a viable therapeutic approach for patients with platinum sensitivity as well as those who have platinum resistance. The rationale behind using PARPis under these conditions, as evidenced by preclinical trials, stems from their capacity to improve the cells’ responsiveness to platinum drugs by impeding their ability to repair damage (Table 1).

**TABLE 1:** Pre-clinical studies.

Authors and year	Type of study	Cell Line	Therapeutic regimen	Type of mutation	Main investigated outcome(s)
Cavallo et al. (2012)	*In vitro*	Embryonal carcinoma cell line (ECs)	Olaparib	Not performed	-EC cell lines have increase PARP1 protein levels and are most likely to respond to PARP inhibitor monotherapy -Olaparib enhance the toxicity of cisplatin in EC cells
Caggiano et al. (2021)	*In vitro*	Cisplatin resistance cell line	Olaparib	Not performed	Pharmacological inhibition of PARP combined with cisplatin had an additive/synergistic effect on cisplatin-resistant cells
Schmidtova et al. (2022)	*In vitro* and *In vivo* (immunodeficient mouse model)	Cisplatin resistance cell line	Veliparib	Not performed	-In vitro the combination of cisplatin with veliparib increased the cytotoxic effect of cisplatin -In vivo analysis not confirm the synergistic effect between PARPi and platinum chemotherapy

Indeed, the synergistic effect of cisplatin and PARPis was initially described in 2012 by Cavallo et al., who reported that in resistant cell lines of embryonal carcinoma (EC) PARPis combined with platinum led to increased DSB formation based on the downregulation of HRR protein expression and a subsequent re-sensitization to cisplatin-induced DNA damage.

Moreover, *in vitro* studies have shown that cisplatin-resistant TGCT cells treated with the PARPis olaparib or veliparib, could be re-sensitized to cisplatin (Cavallo et al., 2012; Schmidtova et al., 2022).

Caggiano C. noticed that the resistance of TGCT cell lines to cisplatin correlates with an increase of efficiency in the DNA repair mechanisms, demonstrating that, due to enhanced DNA repair, cisplatin resistant cells are also resistant to PARPis and that PARPis may interact synergistically with cisplatin. At the basis of the combinatorial effect, they suppose there is a downregulation of HRR protein expression caused by PARPis, with subsequent sensitization to cisplatin (Caggiano et al., 2021).

Another study of Schmidtova et al. evaluated both the possible synergistic effect of PARPis and cisplatin and the expression level of PARP proteins *in vitro* and *in vivo*, showing a high expression of PARP proteins in TGCT cell lines, in particular PARP1 and 2, compared to normal testicular tissue. Therefore, the study supports the data of Caggiano et al., with synergistic effect of veliparib in reversing cisplatin resistance. However, this synergy could not be confirmed *in vivo* using mouse models. This discrepancy highlights the complexity of translating *in vitro* findings to *in vivo* systems, where factors such as drug pharmacokinetics, tumor microenvironment, and adaptive resistance mechanisms can impact on treatment efficacy (Schmidtova et al., 2022).

Another pre-clinical study of Mego et al. confirmed a higher expression of PARP protein in TGCTs in comparison to non-transformed testicular tissue (Mego et al., 2013).

Preclinical studies have indicated that PARP protein expression is elevated in TGCTs compared to normal testicular tissue. However, this elevated expression cannot currently be considered a reliable biomarker for predicting response to PARPis or determining correlation with cisplatin resistance. While these findings suggest a potential avenue for future research, they underscore the need for further investigation to validate PARP expression as a predictive or prognostic marker in TGCTs (Ogino et al., 2010).

## 6 Moving from pre-clinical to clinical studies

De Giorgi et al. assessed the role of Olaparib in a phase II trial as salvage treatment for advanced platinum-refractory TGCTs (two or more prior lines of chemotherapy) (NCT02533765). Primary end point of the study was the overall response rate (ORR). Among the eighteen enrolled patients no partial responses (PR) were observed, and only one of the patients, who was a germline *BRCA1* mutation carrier achieved a stable disease as best response (De Giorgi et al., 2020).

This finding aligns with the preclinical observations of Caggiano et al., who pinpoint that the use of PARPis alone is pointless in platinum-refractory disease due to cross-resistance (Cavallo et al., 2012).

Another phase II study (NCT02401347) of Gruber et al. evaluated the role of talazoparib in 20 patients affected by different types of solid tumors, who received a median of two prior lines of therapy, including platinum-based chemotherapy. The only patient included with TGCT had a germline CHEK2 mutation and achieved a long-term response, highlighting the potential role of PARPis in this rare group of patients and the crucial role of identifying better biomarkers for better selecting TGCT patients for PARPi treatment (Gruber et al., 2022).

**FIGURE 3:**
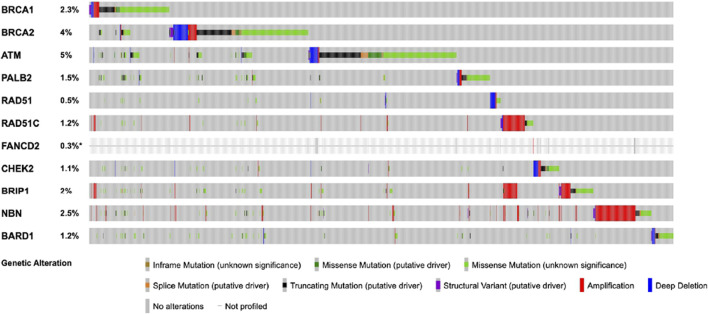
The figure displays the frequency of mutations in homologous recombination repair (HRR) genes in testicular germ cell tumors (TGCTs). The data, obtained from cBioPortal, includes 11,250 samples from 10,635 patients. This combined study contains samples from three studies: MSK-IMPACT Clinical Sequencing Cohort (MSK, Nat Med 2017), Testicular Germ Cell Tumors (TCGA, PanCancer Atlas), and Testicular Germ Cell Cancer (TCGA, Firehose Legacy).

HRD gene mutations, which are pivotal in this selection process, are detected in approximately 5% of testicular cancer cases. This emphasizes the necessity of biomarker-driven approaches to optimize treatment outcomes (Figure 3) (Gao et al., 2013).

In a phase I trial (NCT03318445) by Dhawan et al. the safety and tolerability of pulse dosing and alternate treatment schedules of rucaparib and irinotecan was tested. Fifteen patients with different solid tumors, who had received three or more prior lines of therapy, were included in this study. Three patients with ATM mutations gained control disease for more than 1 year. Unfortunately, no long-term disease control was seen in the 13 patients evaluable for response including one TGCT patients (Dhawan et al., 2020).

Mego et al. evaluated the combination of gemcitabine, carboplatin and veliparib in relapsed/refractory TGCT patients in a phase II trial (NCT02860819). They hypothesized that the inactivation of PARP by veliparib could increase the antitumor activity of gemcitabine and carboplatin in this setting. The study aimed to define the combination’s activity and toxicity. Although the medication was well tolerated, the study’s primary objective was 12-month PFS, which was met by only one (6.7%) of the 15 patients, with a median PFS of 3.1 months. The study did not achieve its primary goal. However, a partial response was identified in four individuals, but that may theoretically be acquired even with the chemotherapy alone, given that carboplatin and gemcitabine are active in TGCTs (Mego et al., 2021) (Table 2).

**TABLE 2:** Phase I/II studies ORR Overall Response Rate, PR partial response, SD stable disease.

Authors and year	Type of study	Line	Therapeutic regimen	N pts	GCT pts enrolled n (%)	Type of mutation	Main investigated outcome(s)
PARPis alone							
De Giorgi et al. (2020)	Phase II	>Second-line treatment (Cisplatin resistant)	Olaparib	18	18 (100%)	Germline and somatic BRCA1-2 mut and BRCA 1–2 wt	ORR **SD: 27.8% SD in BRCA1 mutated patients lasted 4 months
Gruber et al. (2022)	Phase II	>One-line treatment (Cisplatin sensitive)	Talazoparib	20	1 (5%)	BRCA1-2 wt; germline CHEK2 mutation	ORR SD: more than 30 weeks
PARPis plus chemotherapy							
Dhawan et al. (2020)	Phase I	>Second-line treatment	Rucaparib and Irinotecan	15	1 (7%)	Germline or somatic mutation ATM, BRCA1, BRCA2, CHEK2, and PALB2	Overall response rate (ORR):PR rate: 8%
Mego et al. (2021)	Phase II	>Second-line treatment	Gemcitabine, carboplatin and veliparib	15	13 (87%) TGCT 2 (13%) retroperitoneal cancer	Not performed	Overall response rate (ORR) *PR rate: 26.7% and 12-month PFS: 6.7%

## 7 Discussion

This review summarizes the rationale and current evidence on the use of PARPis in cisplatin resistant TGCTs. Thus far, PARPis have failed to show meaningful clinical activity in platinum-refractory TGCT patients, either alone or in combination with chemotherapy. Potential explanations include cross-resistance between platinum compounds and PARPis, the lack of predictive biomarkers or mutations associated with PARPi sensitivity, the intra- and inter-patient heterogeneity of refractory TGCTs and the multifactorial nature of cisplatin resistance.

The evolving landscape of precision medicine holds promise for tailoring treatments to individual patient profiles, optimizing outcomes and minimizing the overall toxicity associated with cancer therapies. In the pursuit of precision medicine, therapeutic agents like PARPis have been evaluated both in preclinical and clinical studies. These inhibitors have been explored not only in monotherapy but also in combination with chemotherapeutic agents. The exploration of combination therapies to enhance treatment efficacy and potentially overcome resistance represents a significant progress in advancing personalized cancer care, although these approaches have not yet proven effective in TGCTs.

*In vitro* experiments suggest that inhibition of different signaling pathways, through PARPis, could lead to restoration of platinum sensitivity in refractory TGCTs, showing a powerful synergistic activity of PARPis with cisplatin (Országhová et al., 2022).

Nevertheless, clinical trials have so far failed to recapitulate these additive effects in patients.

To enhance our understanding and predict the response to PARPis it is crucial to identify specific biomarkers such as the expression level of PARP protein could suggest a possible response to PARPi and different methylation levels of some target gene, in particularly BRCA1 or RAD51, could suggest a potential response to PARPis (Cavallo et al., 2011; Caggiano et al., 2021; Schmidtova et al., 2022).

Moreover, as already acknowledged, there are limited data for the association of TGCTs with hereditary cancer genes (Ottaviano et al., 2021). In the scarce available data, however, the germline mutations detected in TGCTs are in high penetrance DDR genes, such as *BRCA1/2*, MMR gene, *CHEK2* (Ramamurthy et al., 2020). Indeed, the few responses to PARPis in TGCTs were detected in patients with BRCA1/2, ATM or CHEK2 mutations (De Giorgi et al., 2020; Dhawan et al., 2020).

These findings reinforce the idea that patients should be optimally selected for tailored targeted treatments.

## 8 Conclusion

The potential role of PARPis in the context of cisplatin resistance requires further and more in-depth exploration in alignment with the finding of recent studies, as PARP inhibition so far yielded discouraging results in a very limited number of clinical trials. The identification of predictive factors could help the tailored treatment strategy to apply in the poor prognosis patients with refractory/resistant GCTs.
